# Complications and related risk factors of transradial access cannulation for hemodynamic monitoring in general surgery: a prospective observational study

**DOI:** 10.1186/s12871-023-02168-z

**Published:** 2023-06-30

**Authors:** Qin Hou, Bin Zhou, Juanjuan He, Xueying Chen, Yunxia Zuo

**Affiliations:** 1 Department of Anesthesiology, West China Hospital of Sichuan University, 37 Guoxue Alley, Wuhou District, Chengdu, Sichuan 610041 P.R. China; 2 Department of Anesthesiology, Jiangsu Integrated Traditional Chinese and Western Medicine Hospital, Nanjing, Jiangsu Province 210028 P.R. China; 3 Department of Anesthesiology, Chengdu Shang Jin Nan Fu Hospital, Chengdu, Sichuan Province, 611730 P.R. China

**Keywords:** Arterial cannulation, Complications, Transradial access cannulation

## Abstract

**Purpose:**

To examine the short-term complications of arterial cannulation for intraoperative monitoring and their related risk factors.

**Methods:**

We included adult inpatients (≥ 18 years old) who underwent an initial transradial access (TRA) cannulation and were scheduled for general surgery between April 8 and November 30, 2020. We used 20G arterial puncture needles for puncturing and manual compression for hemostasis. Demographic, clinical, surgical, anesthetic, and laboratory data were extracted from electronic medical records. Vascular, neurologic, and infectious complications of TRA cannulation were recorded and analyzed. Logistic regression analyses were used to identify risk factors related to TRA cannulation for intraoperative monitoring.

**Results:**

Among 509 included patients, 174 developed TRA cannulation-related complications. Puncture site bleeding/hematoma and median nerve injury were observed in 158 (31.0%) and 16 (3.1%) patients, respectively. No patient developed cannula-related infections. Logistic regression analysis revealed increased odds of puncture site bleeding/hematoma in women (odds ratio 4.49, 95% CI 2.73–7.36; *P* < 0.001) and patients who received intraoperative red blood cell (RBC) suspension transfusion ≥ 4U (odds ratio 5.26, 95% CI 1.41–19.57; *P* = 0.01). No risk factors for nerve injury were identified.

**Conclusion:**

Bleeding/hematoma were a common complication of TRA cannulation for intraoperative hemodynamic monitoring during general surgery. Median nerve injury may be an under recognized complication. Female sex and extensive intraoperative RBC transfusion are associated with an increased risk of bleeding/hematoma; however, the risk factors for nerve injury remain unclear.

**Trial registration:**

The study protocol was registered at https://www.chictr.org.cn (ChiCTR1900025140).

## Background

Arterial cannulation is widely used for continuous hemodynamic monitoring and arterial blood gas sampling in the operating room and intensive care unit (ICU) [1]. In 1990, the number of arterial catheters placed for direct hemodynamic monitoring was estimated to be 2.5 million in Europe and 8 million in the United States [2]. Increasingly older and medically complex patient populations, together with a growth in the complexity of surgical procedures, have likely led to an increase in the perioperative use of this procedure. In our hospital, the number of direct arterial cannulation is estimated to be more than 20,000 annually. Further, transradial access (TRA) cannulation is most commonly used in general anesthesia surgery. Similar to other invasive procedures, radial artery catheterization involves intrinsic complications.

The complications of TRA include vascular complications such as bleeding/hematoma, compartment syndrome, pseudoaneurysm, radial artery occlusion (RAO), and catheter-associated infection [3–7]. These complications mainly occur in TRA performed for cardiac catheterization, coronary angiography, and percutaneous intervention, which usually involve larger sheath and cannula sizes [8, 9]. Additionally, the use of heparin and antiplatelet drugs, coagulation dysfunction, and hemostasis methods for removing the arterial catheter are crucial factors related to complications of TRA cannulation in cardiac surgery and catheterization [10–13]. However, the incidence of complications of TRA for intraoperative hemodynamic monitoring and their related risk factors remain unclear.

Accordingly, this study aimed to describe the categories and incidences of major short-term complications related to arterial cannulation for intraoperative monitoring as well as to analyze the related risk factors.

## Methods

### Study design

Prospective observational study.

### Setting

This study was conducted at West China Hospital of Sichuan University. The attending anesthesiologist and resident were responsible for TRA cannulation and hemodynamic monitoring.

### Ethical aspects

The study was conducted in accordance with the Declaration of Helsinki, and the protocol was approved by the Ethics Committee of West China Hospital of Sichuan University (number 353, year 2019) and was registered at https://www.chictr.org.cn (ChiCTR1900025140, 13/08/2019). All patients provided written informed consent before enrollment.

### Population

We included patients aged ≥ 18 years who underwent initial TRA cannulation in the operating room before elective general surgery. The exclusion criteria were as follows: 1) language and consciousness barriers as well as inability to communicate; 2) severe coagulation dysfunction was defined according to the diagnostic criteria of DIC by the Scientific and Standardization Committee (SSC) of the International Society for Thrombosis and Hemostasis in 2001, which used PT, platelet count, fibrinogen and D-dimer for integration [14]. In our research, score more than 5 was defined as severe coagulation dysfunction; 3) pre-existing risk of nerve injury such as pain, numbness, paresthesia or dyskinesia in the puncture side limb; and 4) planned postoperative admission to the ICU with the arterial cannula unremoved. Dropout criteria: 1) failed TRA cannulation or altering puncture artery; 2) bleeding/hematoma occurred at the time of puncture; and 3) unplanned ICU admission with the arterial line unremoved.

### Sample calculation

We analyzed 21 preoperative and intraoperative variables. A logistic regression model was used to analyze relevant risk factors. To analyze each variable needing 20 patients, 420 patients were needed. To account for an estimated 20% loss of follow-up, puncture failure, altering puncture artery, and unplanned ICU admission, 504 cases were calculated. Furthermore, considering that the incidence of complications of radial artery puncture might be low, we increased the sample size.

### TRA procedure and data collection

Radial cannulation was performed using 20G arterial puncture needles (Terumo Medical, FS2051) under general or local anesthesia, followed by continuous invasive hemodynamic monitoring. The styloid process of radius was taken as the puncture landmarks. Based on radial styloid process, move about 1 cm to the ulnar side, then 0.5 cm to the proximal cardiac end as the puncture site. The methods used for radial artery puncture, including double-wall puncture, single-wall puncture and ultrasound navigation, were all allowed. All surgeries were performed under general anesthesia with endotracheal intubation; additionally, a cocktail consisted of 0.9% sodium chloride and 5 IU/ml heparin was to keep radial artery access patency in all subjects. Ten minutes after the patient woke up and the endotracheal tube was removed, the radial artery cannula was then removed in the operative room. Subsequently, the site of radial artery cannulation was manually compressed for 10 min using sterilized gauze, and then gauze was placed and tape was applied for another 30 min fixation in the post-anesthesia care unit (PACU). The experimental data collector was not involved in the arterial management process. Once the PACU discharge criteria were met, the patients were transferred to the ward.

### Outcomes and follow-up

#### Complications of radial artery cannulation

The complications were categorized as vascular, neurologic, or infectious complications.

##### Vascular complications

We assessed the occurrence of bleeding/hematoma, distal limb ischemia, symptomatic arterial occlusion, and pseudoaneurysm. Bleeding/hematoma was categorized as grade I (maximum diameter ≤ 5 cm), grade II (≤ 10 cm diameter), grade III (> 10 cm diameter, without reaching the elbow), grade IV (bleeding/hematoma extending beyond the elbow), and grade V (any bleeding/hematoma with an ischemic hand injury) [15, 16]. RAO refers to thrombotic occlusion of the radial artery lumen, initially at the puncture site [17, 18]. Distal limb ischemia and RAO have common clinical symptoms including skin color change, attenuated pulse, cold and pain in the ipsilateral hand. The occurrence of limb ischemia and RAO was preliminarily determined based on the aforementioned clinical manifestations and confirmed by ultrasound when necessary. The clinical symptoms of pseudoaneurysm include pulsatile mass, palpable tremor, and murmur detected in the puncture area, which can be further confirmed by ultrasound or computed tomography [5, 19].

##### Neurological complications

Newly occurring numbness, pain, tingling, stiffness, less power, paresthesia, dyskinesia or loss of function of the ipsilateral forearm and distal hand [20], which will be confirmed by physiologic testing when necessary.

##### Infectious complications

Local infections were identified through local redness, swelling, pain, or secretion at the puncture site and along the cannulation, which will be further confirmed by catheter tip culture. Catheter-related blood stream infections were identified according to the resources of the Infection Prevention and Control Committee based on the Centers for Disease Control and Prevention guidelines [21]. If the same pathogenic bacteria are detected in blood culture with that of catheter tip culture, catheter related infection was confirmed.

### Postoperative follow-up

The first follow-up visit was conducted in the ward 1–3 postoperative days (POD). The second and third follow-up telephone interviews were conducted on 13–16 days and 28–33 days, respectively. When any procedure-related upper extremity complaints related to vascular, neurologic and infectious complications were detected in the first in-person follow-up, patients were asked to describe change of the complaints through telephone interview when discharged.

### Risk factors for the development of TRA complications

The risk factors for TRA complications were chosen based on previous reports mainly focused on transradial coronary angiography and/or a biologically possible hypothesis for the risk of increased injury [1, 13, 15]. We selected the following potential risk factors: age; sex; procedure duration; body mass index (BMI); smoking within the last 1 year; drinking > 2 times/day for 2 weeks; spontaneous activity status; preoperative comorbid diseases, including diabetic status, hypertension, and acute renal failure; hemodialysis; coagulation disorders; heart failure within 30 days; myocardial infarction within 6 months; radiotherapy/chemotherapy within 30 days; weight loss > 10% within 6 months; blood transfusion within 72 h; sepsis within 48 h; disseminated tumor; primary laboratory tests including international normalized ratio (INR), prothrombin time (PT), activated partial thromboplastin time (APTT), hematocrit, platelet (PLT), albumin, glucose, and creatinine.

### Statistical analyses

Statistical analyses were performed using the Statistical Package for the Social Sciences (SPSS), version 22.0. For summary statistics, categorical variables are presented as numbers (n) and percentages (%), while continuous variables are presented as mean with standard deviation (mean ± SD) or median with 25%–75% interquartile ranges. Mann–Whitney U test, χ^2^ test, or Fisher’s exact test were used to compare intergroup differences between patients with and without complications where appropriate. Logistic regression analyses were used to assess risk factors for TRA complications. The complications of TRA cannulation were assessed dependent variables, and the risk factors were evaluated independent variables for the logistic regression model. Additionally, the selected risk factors (independent variables) were assessed linearity before including them in the logistic regression model. The results of logistic regression analyses were summarized using the point estimate for the odds ratio (OR) and 95% confidence interval. For logistic regression model, we included the variables with *P* < 0.1 from the intergroup analysis, and excluded variables if the number of events was too small to calculate the ORs.

## Results

**Fig. 1:**
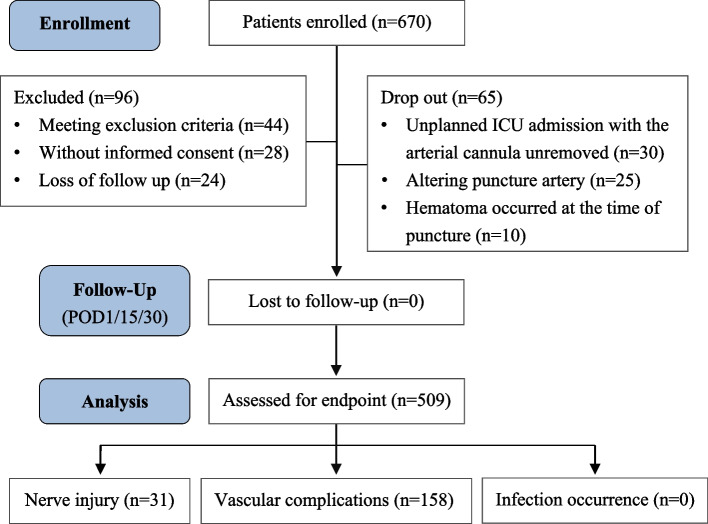
Flow diagram detailing eligible patients

From April 8 to November 30, 2020, 670 patients were initially screened. Finally, 509 patients were assessed for the endpoint (Fig. 1). Vascular, neurologic, and infection complications are described in detail in Table 1. The overall incidence of TRA complications was 34.1% (174 of 509), with the incidence rates of vascular and neurologic complications being 31.0% (158 of 509) and 3.1% (16 of 509), respectively. No catheter-related infections were observed.

**Table 1:** Complications of TRA cannulation in general surgery patients (*n* = 509)

Outcomes	Incidence, n (%)
Vascular complications	158 (31.0)
Hematoma	158 (31.0)
Grade I (0–5 cm)	93 (59.3)
Grade II (5–10 cm)	61(38.6)
Grade III (> 10 cm)	4 (2.5)
Distal limb ischemia	0
Symptomatic arterial occlusion	0
Pseudoaneurysm	0
Neurologic complications	16 (3.1)
Infection complications	0

The most commonly vascular complication was bleeding/hematoma at the puncture site (33.0%, 158/509); among them, the proportions of grade I and grade II hematomas were 59.3% (93 of 158) and 38.6% (61 of 158), respectively. The maximum hematoma diameter in four patients exceeded 10 cm without reaching the elbow (grade III). Further, 28 patients were followed up for the duration of the hematoma, which generally lasted for a median of 15 days (interquartile range, 7–20 days). No other vascular complications were observed.

Nerve injury symptoms were observed in 16 patients. The primary manifestations were numbness and pain of two and a half fingers on the radial side, which pointed to median nerve injury. Symptoms of nerve injury occurred within 3 days after surgery. The duration of neurological injury could be determined in 10 patients. The duration of numbness ranged from 3 h to 30 days, with a median duration of 15 days (interquartile range, 5.75 to 30). There was no dyskinesia or muscle weakness in the area innervated by the median nerve.

### Risk factors for developing bleeding/hematoma

Female patients were more likely to develop hematoma than male patients (Table 2). Intraoperative red blood cell (RBC) transfusion ≥ 4U, longer cannula indwelling time, lower BMI, lower platelet count, and smoking within the last 1 year were also associated with bleeding/hematoma (Table 2).

**Table 2:** Baseline characteristics of patients with and without hematoma

Variables	Hematoma (*n* = 158)	No hematoma (*n* = 351)	Z/χ^2^	*P* value
Age (years)	55 (47, 65)	56 (49, 66)	0.97	0.33
Female	80 (50.6)	76 (21.6)	43.10	< 0.001
BMI (kg/m^2^)	22.5 (20.6, 24.4)	23.1 (21.3, 25.5)	2.93	0.003
Diabetes mellitus	12 (7.6)	44 (12.5)	2.71	< 0.10
Hypertension	23 (14.6)	69 (19.7)	1.91	0.16
Smoking within last 1 year	40 (25.3)	122 (34.8)	4.47	0.03
Laboratory test				
PT (s)	11.2 (10.7, 11.8)	11.3 (10.7,11.9)	0.50	0.61
APTT (s)	27.8 (26.4, 29.8)	28.2 (26.4, 29.9)	0.95	0.51
Hematocrit (%)	40 (37, 43)	40 (37, 44)	0.52	0.59
PLT (× 10^9^/L)	163 (124, 202)	173 (132, 234)	2.15	0.03
Type of surgery				
Liver	84 (53.2)	163 (46.4)	3.02	0.55
Gastrointestinal	44 (27.9)	121 (34.5)		
Pancreas	16 (10.1)	31 (8.8)		
Biliary	7 (4.4)	16 (4.6)		
Other	7 (4.4)	20 (5.7)		
ASA status				
II	121 (76.6)	261 (74.4)	0.28	0.59
III	37 (23.4)	90 (25.6)		
Intraoperative RBC transfusion ≥ 4U	7 (4.4)	4 (1.4)	4.27	0.03
Cannula indwelling time, h	5.2 (4.0, 6.2)	4.7 (3.8, 5.8)	2.18	0.02

Data are median (IQR), or n (%). *P* values were calculated by Mann–Whitney U test, χ^2^ test, or Fisher’s exact test, as appropriate. *BMI* body mass index, *PT* prothrombin time, *APTT* activated partial thromboplastin time, *PLT* platelet, *ASA* American Society of Anesthesiologists, *RBC* Red blood cell

Finally, seven variables were included in the logistic regression model (Table 3). Female sex (OR = 4.49, *P* = 0.001) and intraoperative RBC transfusion ≥ 4U (OR = 5.260, *P* = 0.013) were associated with an increased risk of developing bleeding/hematoma. BMI (OR = 0.914, *P* = 0.008) and PLT level (OR = 0.995, *P* < 0.001) were associated with a decreased risk of developing bleeding/hematoma. There was no significant association between cannula indwelling time and smoking in the last 1 year (Table 3).

**Table 3:** Risk factors associated with hematoma

Risk Factor Variable	Odds Ratio (95% CI)	*P* Value
Female (ref = male)	4.49 (2.73—7.36)	< 0.001
BMI (kg/m^2^)	0.91 (0.85—0.97)	< 0.01
Platelet count (× 10^9^/L)	0.995 (0.992—0.998)	< 0.001
Intraoperative RBC transfusion ≥ 4U	5.26 (1.41 -19.57)	0.01
Diabetes	0.67 (0.33—1.37)	0.28
Cannula indwelling time, h	1.00 (0.99—1.00)	0.23
Smoking within the last 1 year	1.16 (0.69—1.95)	0.55

*BMI* body mass index, *RBC* red blood cell

### Risk factors for nerve injury

Table 4 presented the associations of clinical risk factors with nerve injury. The patients with male gender, younger age, and elevated Hct levels were more probably to experience nerve injury (Table 4). The proportion of male patients with nerve injury was higher than that of male patients without nerve injury (*P* = 0.029). Patients with nerve injury had a lower median age than patients without nerve injury (49 vs. 56 years, *P* = 0.026). Patients with nerve injury had a significantly higher Hct level than patients without nerve injury (43.5% vs. 40%, *P* = 0.039). There were no significant between-group differences in the BMI, diabetes, blood glucose level, smoking history, hypertension, PT, APTT, PLT, ASA status, intraoperative RBCs transfusion ≥ 4U, and arterial cannula indwelling time (*P* > 0.05). The main surgery performed in both groups was hepatic surgery, with no significant between-group difference (*P* = 0.068). Logistic analysis did not reveal risk factors for nerve injury (Table 5).

**Table 4:** Baseline characteristics of patients with and without nerve injury

Variables	Nerve injury (*n* = 16)	No nerve injury (*n* = 493)	Z/χ^2^	*P*
Age (years)	49 (45, 55.5)	56 (48, 65)	2.22	0.02
Male	15 (93.7)	338 (68.6)	3.51	0.02
BMI (kg/m^2^)	22.5 (21.1, 23.8)	22.8 (21.0, 25.3)	0.86	0.39
Diabetes mellitus	1 (6.3)	55 (11.2)	0.38	0.99
Hypertension	3 (18.8)	89 (18.1)	0.005	0.99
Smoking within last 1 year	4 (25)	158 (32.1)	0.35	0.78
Laboratory test				
PT (s)	11.6 (10.8, 12.3)	11.2 (10.7, 11.9)	1.49	1.34
APTT (s)	28.7 (27.2, 29.7)	28.0 (26.4, 29.9)	1.09	0.27
Hematocrit (%)	43.5 (39.5, 47.0)	40.0 (37.0, 44.0)	2.06	0.03
Platelet count (× 10^9^/L)	163 (132.5, 194.5)	170 (132.0, 226.0)	0.92	0.35
Glucose (mmol/L)	5.19 (4.5, 5.8)	5.05 (4.6, 5.8)	0.06	0.95
Type of Surgery				
Liver	13 (81.3)	234 (47.5)	7.56	0.06
Gastrointestinal	1 (6.3)	164 (33.3)		
Pancreas	1 (6.3)	46 (9.3)		
Biliary	0 (0)	23 (4.7)		
Other	1 (6.3)	26 (5.3)		
ASA status				
II	12 (75)	370 (75.1)	0.001	0.99
III	4 (25)	123 (24.9)		
Intraoperative RBC transfusion ≥ 4U	1 (6.3)	11 (2.2)	1.08	0.32
Cannula indwelling time, h	5 (3.9, 6.2)	4.8 (3.8, 5.9)	0.67	0.50

Data are median (IQR) or n (%). *P* values were calculated by Mann–Whitney U test, χ^2^ test, or Fisher’s exact test, as appropriate. *BMI* body mass index, *PT* prothrombin time, *APTT* activated partial thromboplastin time, *ASA* American Society of Anesthesiologists, *RBC* red blood cell

**Table 5:** Risk factors associated with nerve injury

Risk Factor Variable	Odds Ratio (95% CI)	*P* Value
Male (ref = female)	0.13 (0.02, 1.04)	0.05
Age, years	0.96 (0.92, 1.01)	0.12
Type of surgery	-	0.77
Hematocrit (%)	0.99 (0.90, 1.10)	0.92

## Discussion

This single-center prospective study described the incidence of major complications of TRA cannulation for perioperative hemodynamic monitoring in adult patients. The major complications were bleeding/hematoma and nerve injury. The risk factors for bleeding/hematoma included female sex and intraoperative RBC transfusion ≥ 4U. The most common nerve injury was median nerve injury. However, the risk factors for nerve injury remain unclear.

Several clinical reviews have summarized postprocedural complications of hemodynamic monitoring using radial arterial catheters in anesthesia and intensive care medicine, as well as TRA for percutaneous coronary intervention [7, 12, 13]. Temporary RAO is the most commonly postprocedural vascular complication [7]. There have been case reports of rare complications, including abscess, median nerve paralysis, suppurative thrombarteritis, air embolism, compartment syndrome, and carpal tunnel syndrome [22–26]. Bleeding/hematoma at the puncture site is another common complication of radial cannulation (incidence rate: 14%), regardless of whether it is for hemodynamic monitoring or interventional cardiac procedures [13]. A recent study reported that the incidence of bleeding/hematoma at the percutaneous radial artery access site for peripheral vascular interventions was 4.8% [27]. There are several hemostatic compression practices, which range from manual compression to compression dressings; further, they have been a recent increase in the use of circumferential bands capable of applying titratable pressure [28]. A study using a patent radial compression device (TR Band®) reported that the incidence of bleeding/hematoma during transradial percutaneous cardiovascular procedures was 10.2% [4]. However, the incidence of bleeding/hematoma after TRA with local manual compression remains unclear.

We found that bleeding/hematoma is the major vascular complication of TRA for monitoring purposes with local manual compression. In our research, TRA cannulation was used for invasive blood pressure monitoring, arterial blood gas analysis, and fluid resuscitation guidance. No intravascular procedures were performed through the arterial cannula. Therefore, there was significantly less intimal injury to the radial artery. Local manual compression is generally effective for achieving hemostasis. The firm and flat base of the radial bone as well as the thick-walled structure of the radial artery increase the suitability for compression; accordingly, hemostasis can be achieved by applying modest pressure. However, given the variations in the anatomical position of the radial artery at the wrist [29, 30] and the margin of safe distance from the wrist for needle puncture of a radial artery (5.4 cm in women and 6.8 cm in men) [11], it is difficult to accurately compress the proximal end of the puncture site during manual compression. Accordingly, the effectiveness of hemostasis cannot be ensured. Moreover, after removing the arterial cannula, 10 min of manual compression was initially applied over the gauze placed on the puncture site. Subsequently, 30 min of compression bandage was wrapped around the wrist over the puncture site. Therefore, the compression strength could not be standardized and quantified. Based on clinical practice and relevant literature [31], the compression time was set to 40 min; however, it remains unclear whether this time was sufficient for achieving hemostasis. Taken together, the aforementioned factors could have contributed to the ineffectiveness of traditional manual compression and may explain why bleeding/hematoma was the most common complication in our study.

Reported risk factors for puncture-related complications after cardiac interventions mainly include age, sex, sheath size, use of heparin and antiplatelet drugs, and coagulation dysfunction. Additionally, the hemostasis method used to remove the arterial cannula is a crucial factor for complications of radial artery catheterization in cardiac surgeries and cardiac catheterization [10–13]. Therefore, we examined whether the aforementioned risk factors can predict complications of TRA for monitoring purposes. Since we included patients who underwent general surgery, with most of them not taking any anticoagulants or thrombolytic agents, we identified some different risk factors. Female sex and intraoperative RBCs transfusion ≥ 4U were significant risk factors for bleeding/hematoma. Consistent with our findings, Dale et al. reported that compared with male patients, female patients had a higher incidence of local adverse vascular events, which could be attributed to smaller blood vessels in women as well as other anatomical and hormonal sex differences [32]. Additionally, we found that intraoperative RBC transfusion of ≥ 4U increased the risk of bleeding/hematoma. Patients who require intraoperative blood transfusion usually experience extensive bleeding and blood loss. Moreover, extensive bleeding and blood transfusion can result in coagulation dysfunction such as dilution coagulopathy [33], which further increases the risk of bleeding or hematoma.

Notably, we found that 3.1% of the patients had complaint of numbness and pain in the innervated area of the median nerve, with the symptoms being temporary. Although studies have shown that nerve injury is a rare complication of radial artery puncture [20, 34, 35]. Maarten A.H et al. also reported a high incidence of percutaneous coronary procedures-related upper extremity complaints through the radial artery including pain (43%) and numbness (7%) [36]. The median nerve can be affected by acute trauma, chronic microtrauma, and compressive lesions [37]. Various mechanisms could contribute to median nerve damage. First, prolonged hemorrhage at the puncture site may result in hematoma expansion; however, there was no evidence of nerve injury related to bleeding/hematoma (data not shown). The pattern suggested that hemorrhage-induced neuropathy resulted from a combination of direct nerve compression and focal nerve ischemia caused by vasa nervorum compression [38]. However, since we did not perform ultrasound or neurophysiological examinations, we could not determine whether the nerve injury was caused by incorrect gauze compression or hematoma. Second, direct nerve damage can result from the inappropriate placement of cannula tips, which is crucially influenced by the operator’s experience. Third, nerve damage may result from nerve ischemia caused by varying degrees of arterial thrombosis. The methods used to achieve hemostasis may be relevant [34, 35] even though this was not a significant factor in our study. Finally, wrist hyperextension for arterial cannula placement and stabilization could cause profound impairment of median nerve function [39]. Since the operators who performed radial artery punctures underwent standardized training, this factor could have been eliminated in our study.

The study has several limitations. First, we did not standardize the methods used for radial artery cannulation, including double-wall puncture, single-wall puncture, and ultrasound-guided puncture. Our research aims to explore the complications of radial artery catheterization in the real world. Therefore, we do not unify the puncture method. As a result, repeated punctures may contribute to the high incidence of bleeding/hematoma and nerve injury. Second, the outcome indicators were mainly defined by clinical symptoms; therefore, several complications may have been underestimated or missed. Since we could not accurately evaluate all factors regarding the puncture, including repeated punctures, the results of the logistic regression model may be biased. Third, venous and artery access may have been on the ipsilateral limb in some cases, which may also have led to an overestimation of the incidence of TRA-related bleeding/hematoma.

## Conclusions

To our knowledge, this is the largest prospective study on the complications of TRA cannulation during intraoperative hemodynamic monitoring, with the most common complication being bleeding/hematoma. Traditional manual compression may be ineffective in achieving hemostasis. Further, median nerve injury may be an underestimated complication of TRA cannulation and further studies are warranted. Female sex and extensive intraoperative RBCs transfusions were risk factors for the development of bleeding/hematoma; however, the risk factors for nerve injury remain unclear.

## Data Availability

The datasets used and/or analyzed during the current study are available from the corresponding author on reasonable request.

## Abbreviations

APTT: Activated partial thromboplastin time
BMI: Body mass index
CI: Confidence interval
DIC: Disseminated intravascular coagulation
Hct: Hematocrit
ICU: Intensive care unit
PACU: Post-anesthesia care unit
PLT: Platelet
POD: Postoperative day
PT: Prothrombin time
RAO: Radial artery occlusion
RBC: Red blood cell
TRA: Transradial access
